# Application of Machine Learning Strategies to Model the Effects of Sevoflurane on Somatosensory-Evoked Potentials during Spine Surgery

**DOI:** 10.3390/diagnostics13213389

**Published:** 2023-11-06

**Authors:** John Preston Wilson, Deepak Kumbhare, Charles Ronkon, Bharat Guthikonda, Stanley Hoang

**Affiliations:** Department of Neurosurgery, Louisiana State University Health Shreveport, Shreveport, LA 71103, USA; jpw002@lsuhs.edu (J.P.W.J.); deepak.kumbhare@lsuhs.edu (D.K.); charles.ronkon@lsuhs.edu (C.R.); bharat.guthikonda@lsuhs.edu (B.G.)

**Keywords:** machine learning, neural networks, principal component analysis, phase space curve analysis, support vector machine, regression learners, time-frequency analysis, intraoperative neuromonitoring, somatosensory evoked potential, sevoflurane

## Abstract

In this study, a small sample of patients’ neuromonitoring data was analyzed using machine learning (ML) tools to provide proof of concept for quantifying complex signals. Intraoperative neurophysiological monitoring (IONM) is a valuable asset for monitoring the neurological status of a patient during spine surgery. Notably, this technology, when operated by neurophysiologists and surgeons familiar with proper alarm criteria, is capable of detecting neurological deficits. However, non-surgical factors, such as volatile anesthetics like sevoflurane, can negatively influence robust IONM signal generation. While sevoflurane has been shown to affect the latency and amplitude of somatosensory evoked potential (SSEP), a more complex and nuanced analysis of the SSEP waveform has not been performed. In this study, signal processing and machine learning techniques were used to more intricately characterize and predict SSEP waveform changes as a function of varying end-tidal sevoflurane concentration. With data from ten patients who underwent spinal procedures, features describing the SSEP waveforms were generated using principal component analysis (PCA), phase space curves (PSC), and time-frequency analysis (TFA). A minimum redundancy maximum relevance (MRMR) feature selection technique was then used to identify the most important SSEP features associated with changing sevoflurane concentrations. Once the features carrying the maximum amount of information about the majority of signal waveform variability were identified, ML models were used to predict future changes in SSEP waveforms. Linear regression, regression trees, support vector machines, and neural network ML models were then selected for testing. Using SSEP data from eight patients, the models were trained using a range of features selected during MRMR calculations. During the training phase of model development, the highest performing models were identified as support vector machines and regression trees. After identifying the highest performing models for each nerve group, we tested these models using the remaining two patients’ data. We compared the models’ performance metrics using the root mean square error values (RMSEs). The feasibility of the methodology described provides a general framework for the applications of machine learning strategies to further delineate the effects of surgical and non-surgical factors affecting IONM signals.

## 1. Introduction

The field of spine surgery has the privilege of integrating cutting-edge technology into daily practice. An example is intraoperative neuromonitoring (IONM), which is a technology frequently used during spine procedures to monitor neurophysiologic parameters in order to detect potential intraoperative injury and allow time for intervention to minimize the development of neurologic deficits [1]. IONM utilizes different channels or modalities to capture and transmit various sources of neurophysiologic data. The data are obtained by applying stimulation to leads connected to the patient, either peripherally or to the skull, depending on the specific pathway being monitored. The primary IONM modalities include somatosensory evoked potential (SSEP), transcranial motor evoked potentials (TcMEPs), and spontaneous or triggered electromyography (EMG) [2,3,4]. Using specific stimulation paradigms designed for optimal data collection during a spine operation, IONM technicians inspect incoming stimulation data for any signal alteration from baseline, which may indicate a neurologic insult from surgical manipulation, non-surgical factors, or not uncommonly, from mechanical artifacts. An example of a non-surgical factor can be seen during patient positioning, which if carried out improperly, can trigger detectable signal alteration that allows the surgeon to immediately re-position the patient to avert any potential deficit [5,6]. Nevertheless, distinguishing surgical from non-surgical factors or mechanical artifacts leading to signal alteration is of paramount importance for proper troubleshooting of IONM waveform disturbances.

A non-surgical factor that has been shown to significantly affect IONM modalities is the anesthetic regimen used during a particular spine operation. Surgeons, anesthesiologists, and IONM technicians must be aware of this finding, as it can have significant implications on IOMN signal validity and directly affect patient outcomes [7]. Specifically, volatile anesthetic agents have been shown to affect robust IONM signal generation [8,9]. This effect has been observed with TcMEPs, with evidence suggesting that evoked responses traveling within polysynaptic pathways are more susceptible to anesthetic agents, resulting in a deviation from the signal baseline. Achieving a deep neuromuscular blockade is not possible during TcMEP monitoring due to the necessary muscular activity needed for measurement, such that a short or intermediate duration of action muscle relaxant is typically utilized [10]. As relevant to this current investigation, SSEPs are also affected by volatile anesthetic agents, yet the investigation into SSEP waveform changes is not as robust as that for TcMEPs. Moreover, the research available on SSEP and volatile anesthesia is limited to quantifying the dose effects of these agents on SSEP waveforms. In addition, recent focus has shifted to optimizing the anesthetic regimen rather than improving the technology that enables the best pharmacologic agents to be used. For example, total intravenous anesthesia (TIVA) has been favored over inhaled anesthetics in recent years due to TIVA’s lower inhibitory effect on SSEP signals [11]. TIVA is an anesthesia protocol that typically pairs an intravenous agent such as propofol with a commonly used opioid such as remifentanil, while eliminating the use of inhaled anesthetics. However, TIVA is not the ideal anesthesia for post-operative evaluation due to the prolonged recovery time, which delays the accurate assessment of the patient’s neurological status [12,13]. However, as previously mentioned, due to the inhibitory nature of volatile anesthetics on SSEP waveforms, low dose agents with a minimum alveolar concentration (MAC) of less than 0.5 and up to 1 are typically used [14]. One study investigating the dose-dependent effects of sevoflurane and desflurane at varying concentrations of MAC found that the amplitudes of SSEP signals were decreased and the latencies delayed with increasing concentrations [15]. Furthermore, recommendations for IONM troubleshooting during SSEP signal changes for correctable causes include searching for the use of halogenated anesthetics [16]. Regardless, it is worth noting that volatile anesthetics such as sevoflurane, desflurane, enflurane, halothane, and isoflurane have a shorter recovery time to allow for faster post-operative neurological evaluation and, just as important, are less expensive when compared to TIVA [17].

Yet, despite these recognized advantages of volatile anesthetics, their effects on IONM signals make this class of medications challenging to use. Specifically, volatile anesthetics can affect SSEP signal amplitude and latency, prompting the need for multi-modal IONM techniques [15,18,19]. The integration of combined SSEP, TcMEPs, and EMG provides a more accurate and multi-angle viewpoint of the functional integrity of the spinal cord, which can enhance surgical precision and reduce the risk of neurological injury [20,21,22,23]. Nonetheless, the massive amount of data generated from multi-modal monitoring makes it difficult to quantify the range of variability seen from these waveforms. As such, machine learning (ML) has been identified as a potential solution to better characterize the range of IONM disturbances affecting signal waveforms [24].

Machine learning (ML) is a subset of artificial intelligence (A.I.) that enables systems to learn from previous data to predict future outcomes. A number of ML models are available, depending on the degree of direct human involvement. Supervised ML models use human-generated labeled data to train and validate algorithms to classify data or predict outcomes [25,26]. Conversely, unsupervised learning ML models are guided very little by their users. These models work independently to identify patterns and trends in datasets that are not easily detectable by humans [27]. This is achieved by using dimensionality reduction techniques to extract meaningful information from seemingly chaotic or random datasets. Dimensionality reduction techniques such as principal component analysis (PCA), time-frequency analysis (TFA), and phase space curves (PSCs) can be used to detect changes to IONM signal waveforms. This study attempts to use machine learning to understand the relationship between a volatile anesthetic agent and SSEP in humans. It aims to investigate the potential of an unsupervised ML technology to model and predict the effects of changing end-tidal sevoflurane concentrations on SSEP waveforms in patients undergoing spine surgery. The successful application of ML strategies to analyze IONM data is the major contribution of this study. To develop fully automated IONM systems, it is necessary to take a methodical approach to identifying sources of signal artifacts. The data included in the study are the first steps towards the development of ML IONM predictive models. Due to the lack of existing literature or methodology applied to neuromonitoring data, our study utilized a small sample of patients for quantifiable proof of concept using ML tools.

## 2. Materials and Methods

### 2.1. Patient Population

This study was approved by the Institutional Review Board (IRB). All patients were consented before their surgeries. Patients who underwent surgical treatment for the pathology of the thoracic and lumbar spine and were 40 years of age and older were included. Patients were excluded if they received surgical treatment for cervical spine pathology. Cases that were administered sevoflurane exclusively as a volatile anesthetic were considered. Furthermore, patients were excluded if they were administered any other volatile anesthetic agent besides sevoflurane or if patients experienced intraoperative difficulties resulting in new deficits. Following patient selection, ten patients (seven men and three women, ages ranged from 49 to 74 years old) who underwent surgeries between January 2023 and August 2023 were entered into the study (Table 1). One surgeon performed the surgeries at a single academic medical center.

### 2.2. Anesthesia Protocol

Following induction of anesthesia and endotracheal intubation, a bite block was placed in the mouth to prevent damage inflicted on the teeth, cheeks, or gums during neuromonitoring. Inspired sevoflurane was administered at varying concentrations during each procedure, and the anesthesiologists decided on any concentration changes to ensure stable hemodynamic parameters.

### 2.3. IONM Protocol

**Figure 1:**
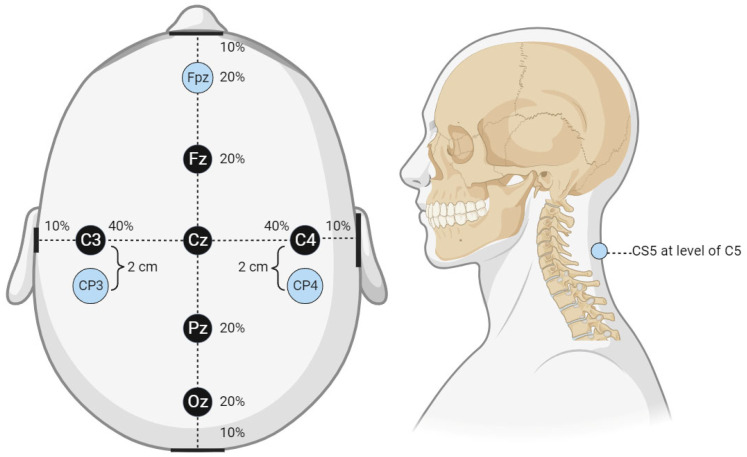
Cortical electrode placement. This figure displays the cortical electrode placement for SSEP signal recording used by the technicians from which waveform data were collected. The percentage values shown in the figure correlate to the approximate distance from the center reference point, Cz. For example, Fz is 20% of the distance from the center reference point to the anterior reference marker (nasal bridge), Fpz is located an additional 20% of the distance from Fz or 40% of the distance from Cz. The same applies to all reference markers and the associated percentages. The lateral reference markers are in line with the center of the ears, while the posterior reference marker is the center of the occiput.

**Figure 2:**
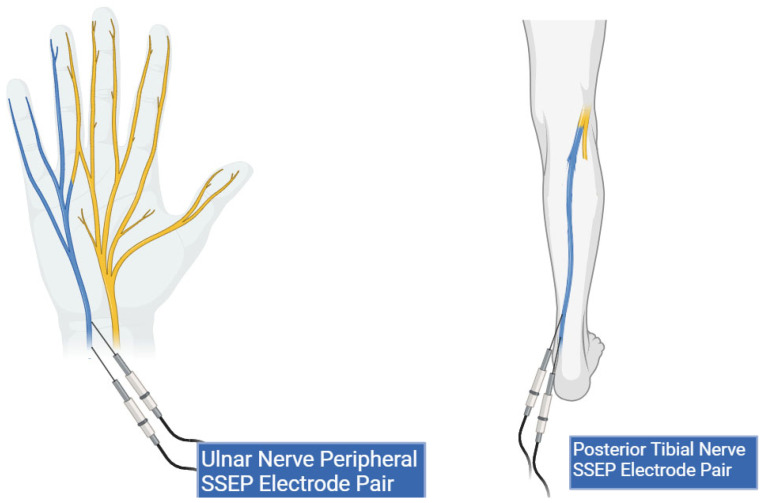
Peripheral lead targets for SSEP monitoring. This figure shows peripheral electrode placement for the ulnar (**left**) and posterior tibial nerves (**right**) (highlighted in blue). Two stimulation electrodes are shown in both peripheral targets representing the cathode and anode pair.

A local third-party provider for neuromonitoring services was used for all cases. All patients were monitored using multiple IONM modalities, including SSEP, EMG, and TcMEPs. The technicians used Cascade Surgical Studio software version 3.5.1680 by Cadwell for streaming and storage of IONM case data. For this study, only SSEP waveforms were extracted from each case. Figure 1 displays the cortical electrodes and Figure 2 peripheral nerve targets for SSEP monitoring. Neuromonitoring SSEPs were collected from the upper extremities using peripheral leads stimulated using a constant current with intensity ranging from 0.025 to 0.034 amperes (A) applied with normal polarity to the left and right ulnar nerves. For the lower extremities, a constant current was applied during stimulation to the left and right posterior tibial nerves with an intensity ranging from 0.04 to 0.06 (A) and normal polarity. SSEP waveforms were recorded using specified electrode pairs attached to each patient’s scalp, with one subcortical lead at the level of C5. The ulnar nerve was targeted for the upper extremities to monitor SSEPs in the right and left arm. The posterior tibial nerve was targeted for the lower extremities to monitor SSEPs in the left and right leg. The three channels that SSEP waveforms were collected from varied slightly depending on the targeted nerve. The channels used to record the left ulnar nerve waveforms consisted of the following montages: CP4 (active body site)–Fpz (reference body site), CP4 (active body site)–CP3 (reference body site), and CS5 (active, subcortical body site)–Fpz (reference body site). For the right ulnar nerve, the channels data collected were CP3 (active body site)–Fpz (reference body site), CP3 (active body site)–CP4 (reference body site), and CS5 (active, subcortical body site)–Fpz (reference body site). For the left posterior tibial nerve, the channel data collected came from CPz (active body site)–Fpz (reference body site), CP3 (active body site)–CP4 (reference body site), and CP3 (active body site)–Fpz (reference body site). Lastly, for the right posterior tibial nerve, the channel data were collected from CPz (active body site)–Fpz (reference body site), CP4 (active body site)–CP3 (reference body site), and CP4 (active body site)–Fpz (reference body site).

### 2.4. Data Collection

Anesthesia data for end-tidal sevoflurane concentrations were recorded by the anesthesia information system (sampling rate: 1 reading/min) and uploaded to the patient’s digital healthcare charts (EPIC) actively throughout the case. These data were manually reviewed, extracted, and entered into a Microsoft Excel spreadsheet for future analysis. End-tidal sevoflurane concentrations were chosen for data analysis over inspired sevoflurane concentrations because they represented the level of volatile anesthesia circulating and expressing its effects throughout the body. IONM case data were exported from the technicians’ recording workstations in a (zipped) file format. Using Cadwell export software, (zipped) case data files were exported in a (.json) file format for further analysis. Time points for end-tidal sevoflurane concentrations and IONM trial data were imported into MATLAB software version R2022a for timepoint synchronization in later stages. De-identification of patient data was completed after successfully importing all desired datasets into the MATLAB.

### 2.5. Feature Generation

Further data processing and analysis were performed in MATLAB (MathWorks v.R2022a). Sourced (.json) files were converted into MATLAB (.mat) datafile format. SSEP data were identified and exclusively extracted from each trial. SSEP waveforms from different trials were sorted into new variables based on their respective nerve–channel pairing (e.g., left ulnar nerve: CP4–Fpz). Following the sorting of SSEP waveforms, feature generation metrics were developed as follows:

(1) Phase Space Curve. The Phase Space Curve is recognized as a mathematical tool for plotting a solution to a set of equations that describe the motion of the phase plane or phase flow through space [28]. It is used to help process data that trend towards chaos by generating a three-dimensional (3D) geometric shape representative of the data points within a set timeframe.
(2) Evoked Response Latency. This data feature is commonly used to describe SSEP waveforms for the first two channels of the ulnar nerve. The N20 (negative peak at 20 milliseconds following stimulation) and P30 (positive peak at 30 milliseconds following stimulation) values relay information about the latency of evoked responses. The N13 (negative peak at 13 milliseconds following stimulation) and P14 (positive peak at 14 milliseconds following stimulation) values are used for the third channel of the ulnar nerve. For the left and right posterior tibial nerves, P37 (positive peak at 37 milliseconds following stimulation) and N45 (negative peak at 45 milliseconds following stimulation) values were assessed for all three channels.
(3) Evoked Response Amplitude. Data for evoked response amplitudes correlate to the height of signal waveforms at the latency time points. These values were extracted from SSEP waveforms only for the time points of interest.
(4) Time-Frequency Analysis. This method translates information from two domains in one dimension (time domain and frequency domain) into one domain in two dimensions [29]. It enables the analysis of these two domains simultaneously, allowing users to analyze the more commonly recurring signal frequencies within a set amount of time. In TFA, the peak or dominant frequency is the greatest frequency value recorded for any signal within a given time segment. The power at the peak frequency is another feature extracted from TFA spectrograms. Lastly, the timestamp of the intercept for peak frequency and power provides information about when this response occurred.
(5) Principal Component Analysis. This technique is useful for reducing the variables associated with large datasets. Reducing the number of variables, or principal components, to the minimum amount afforded allows for retaining most of the signal waveform information and prompts for more robust signal clustering [30].

**Figure 3:**
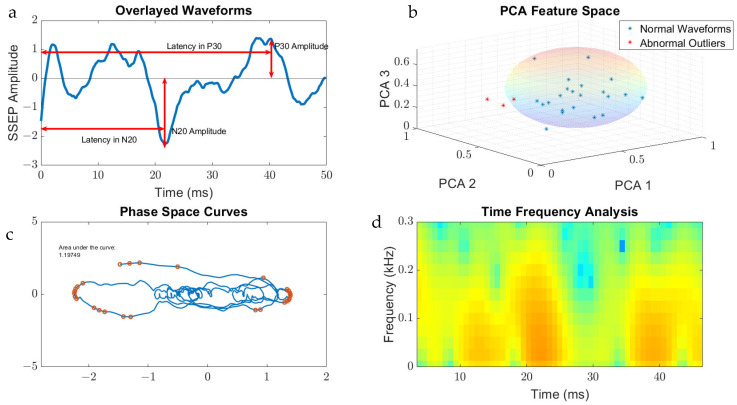
Metric categories for SSEP waveforms. Figure 3 displays the metric categories for which SSEP data were analyzed for feature generation using all the trials from one patient as an example. (**a**) is a raw SSEP waveform from a left ulnar nerve cortical channel demonstrating the different features of signals. Shown here is the positive peak signal latency, representing the delay associated with the positive peak ‘P30′ occurring at 30 milliseconds following applied stimulation. Similar signal characteristics are applied for the negative latency occurring earlier following applied stimulation. The ‘N20′ latency and amplitude are correlated to the lowest trough value at 20 milliseconds following stimulation. (**b**) is a graph showing the results of SSEP waveform data processed using principal component analysis. Shown in the graph are the first three principal components accounting the for majority of variance found in the different trials for this patient. The baseline waveforms are plotted with blue datapoints, while abnormal waveform trials are shown in red. The sphere demonstrates the clustering of normal waveforms. (**c**) shows a phase space curve connecting the different trial points. This higher dimensional representation of signals allows for comparison between different trials using geometric shapes for calculating the area of the curve. (**d**) is a time–frequency domain spectrogram, plotting signal frequencies and demonstrating the power of these frequencies within a set timeframe. Red areas of the plot are associated with higher powers compared to green and blue areas.

The features that were generated for SSEP waveform analysis were derived from the qualities of the signal processing tool used on the datasets. For example, the area of the phase curve was the feature associated with PSC, while TFA presented data in a format that allowed for interpretation of peak signals and frequencies. Figure 3 displays the metric categories from which features were generated for this study.

### 2.6. Synchronization of Anesthesia and IONM Timepoints

The clock time of the IONM workstation and the clock time of the anesthesia information system were confirmed to be synchronized during the cases. Time points of end-tidal sevoflurane concentrations that occurred during SSEP trials were exclusively identified and extracted. The end-tidal sevoflurane concentrations at these identified time points were assigned as the independent variable.

### 2.7. Normalization of Data

All features within the metric categories generated were normalized using interquartile range normalization (IQR). The IQR is calculated using the difference between the third and first quartiles [31]. Using this method to normalize the data between different features, outliers in the dataset are less likely to affect future predictive models generated.

### 2.8. Reverse Regression Analysis

Following the normalization step, all the data from ten patients were consolidated for regression analysis to identify the features more directly correlated with the changing end-tidal sevoflurane concentrations. The current study involves a single input or independent variable (end-tidal concentration of sevoflurane) and the dependent variable as the SSEP waveforms. Eleven different metric categories were generated to characterize the SSEP waveforms. The goal was to understand how changes in sevoflurane concentration could impact the outcome variables, identified as the 11 SSEP features. We employed reverse regression analysis for this scenario. In the reverse regression analysis, the independent variable (sevoflurane concentration) was flipped to become the dependent and continuous output variable [32,33], while the 11 SSEP features were assigned to serve as the independent or predictor variables. The data were then input into the MATLAB regression learner application for further processing and modeling.

**Figure 4:**
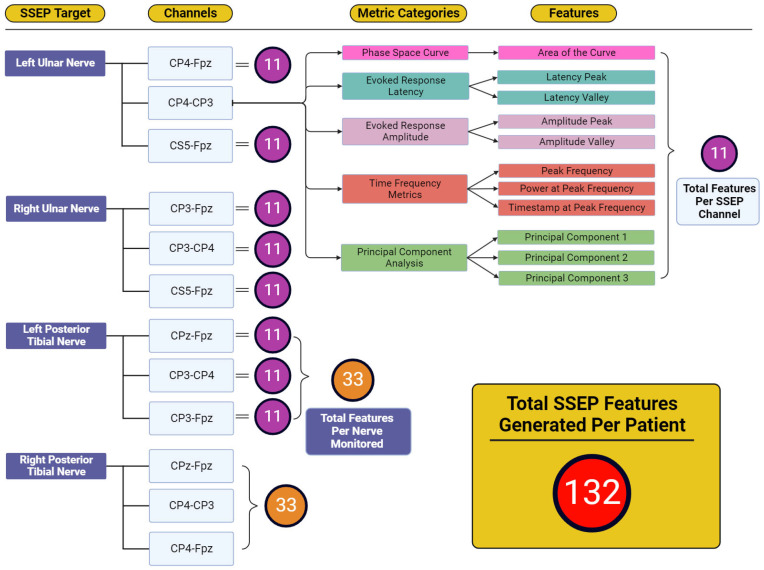
Feature generation diagram for each monitored nerve. This figure shows the framework for how 132 total features were generated for each patient. Each nerve shown in the ‘SSEP Target’ column has three channels from which SSEP signals were recorded. Each of these channels recorded trials that were entered into each of the metric category. Within the metric categories, different features were generated depending on the metric category being analyzed. Shown in the features column were the entirety of features generated from the five metric categories. The total features for each channel totaled 11 features. Eleven features for each channel and thirty-three features between three channels for each of the four nerves monitored was how the one-hundred and thirty-two total features were generated for each patient.

Figure 4 displays the total features representing the output variables for which reverse regression analysis was performed. Five individual metric categories were assessed for each channel used when targeting SSEP for monitoring. These metric categories consisted of phase space curve analysis (one feature—the area of the curve), evoked response latency (two features—latency peak and latency valley), evoked response amplitude (two features—amplitude peak and amplitude valley), time-frequency metrics (three features—peak frequency, power at peak frequency, and timestamp at peak frequency), and principal component analysis (three features—generated from the first three principal components accounting for approximately 95% of waveform shape). The number of features developed totals 11 features for each channel, 33 features for each nerve (three channels per nerve), and 132 features for each patient (four monitored nerves per patient).

### 2.9. Feature Selection

The MATLAB regression learner application was used for feature selection and regression modeling. For feature selection, the minimum redundancy maximum relevance (MRMR) method was used to estimate an important score for each feature. Using the 33 features obtained through the signal processing tools for each channel within each nerve group, the importance of each feature was compared. By analyzing the development of SSEP signals over time and the significance of each feature, we were able to determine the features that were most indicative of changes in IONM signals. The top ten highest features were selected for modeling based on their MRMR importance score.

### 2.10. Regression Modeling

Two patients’ data from the total dataset were set aside for testing the results of the validation metrics performed later. The other eight patients from the data pool were used for training the models. During training stages, ML models and their internal variables, or parameters, were assigned varying weights to fine-tune the models’ predictive capabilities. Cross-validation fold five was used. The data were introduced for the following different models:

(1) Linear Regression. This is a useful model when estimating the association of independent predictor variables, and the continuous output variables are maintained [34].
(2) Regression Trees (fine tree, medium tree, coarse tree). This model encompasses a range of different predictive models that start with the first leaf, or node, and follows a tree pattern to the next leaf containing the value for the response. Fine, medium, and coarse describe different leaf sizes for desired data fitting goals.
(3) Support Vector Machine (linear, quadratic, cubic). SVMs use kernel functions (linear, quadratic, cubic) to determine the transformation applied to datasets before training. Plotting datapoints against a hyperplane in high-dimensional space allow SVM binary classifiers to assign incoming data to the possible output classes [35].
(4) Neural Networks (narrow, medium, wide). Neural networks combine layers of network input at varying sizes (narrow, medium, wide) to feed forward to the next connected layer. Each layer multiplies the network input by a weight matrix, and then assigns a bias vector. The final layer generates the predictive output [36].

**Figure 5:**
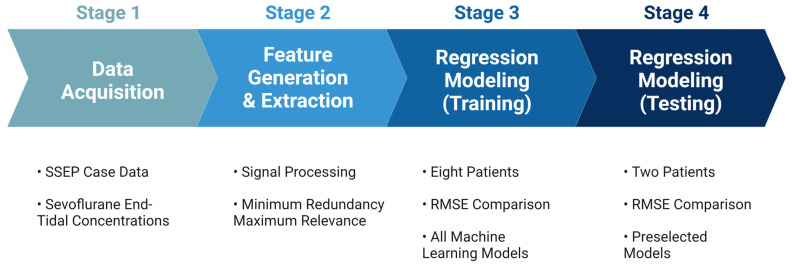
Development for machine learning system. The figure depicts the pathway towards the final ML predictive model used for analyzing SSEP data. Stage 1 comprised of data acquisition of the various datasets that were to be included in the study. Stage 2 was the generation of features used to represent the datasets in different signal processing tools, and the subsequent ranking of those features for their importance. Stage 3 was the training of the ML models that were considered for this study. Lastly, stage 4 was when the models were tested, and performance metrics were compared.

The performance of each model was evaluated based on their associated root mean square error values (RMSEs), which measure the average difference between the values that any given model may predict and the actual data values within a dataset [37]. This value serves as a measure of accuracy to identify which models could assign the most significant predictive value for the features provided to the models. Once all models were trained using the highest performing features identified in each nerve group, the models with the most accurate predictive capabilities were chosen for testing. The highest-performing models were tested on the remaining two patients from the original dataset. Figure 5 shows an overview of the steps taken during the development of the prediction model.

## 3. Results

### 3.1. Patient Details

The primary purpose of this study is to provide a proof of concept for the use of machine learning applications for neuromonitoring data. In light of this, a small sample size of patients was initially selected to be included in the study. The patients included in the study were primarily male, with three female patients. The average age of patients included in the study was 58.8 years old, with a standard deviation of 7.7 years. Eight of the ten patients in the study were surgically treated with lumbar decompression and fusion for compressive symptoms. One patient was treated with thoracic fusion, and one patient had an extradural neoplasm excised in addition to a lumbar fusion. The details of the patients included in the study can be found in Table 1.

### 3.2. Feature Selection for Each Monitored Nerve

**Figure 6:**
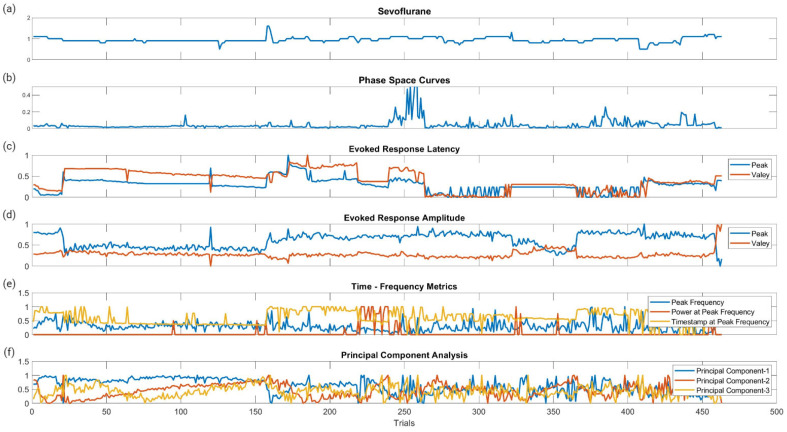
Raw data points of the metric categories for SSEP trials. This figure shows the plotted changes derived from the different metric categories and their associated features shown in Figure 3 for all ten patients. (**a**) is the plotted changes in sevoflurane concentration over the different trials. (**b**) is the plotted changes in the areas of the phase space curve over the different trials. (**c**) is the changes in the evoked responses latency peak and valleys for the different trials. (**d**) is the changes in the evoked responses amplitude peak and valleys for the different trials. (**e**) is the changes for all three features (peak frequency, power at peak frequency, and timestamp at peak frequency) in time frequency analysis signal processing for the different trials. (**f**) is the changes in the first three principal components for the different trials.

**Figure 7:**
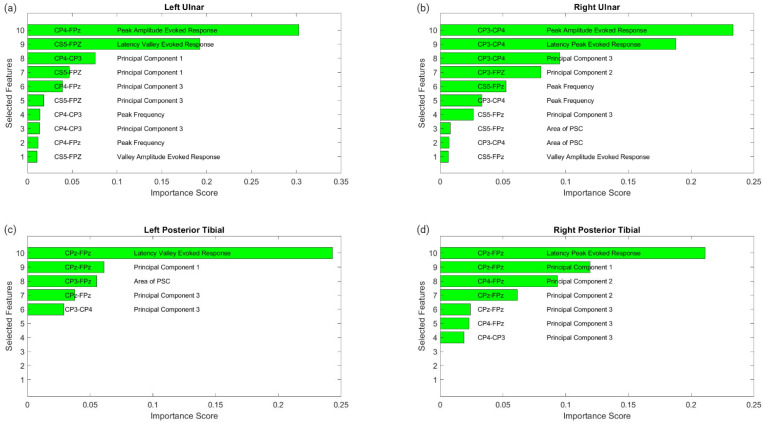
MRMR results for feature selection. This figure shows the results of feature selection for the different nerve categories after performing MRMR to determine which of the 132 features was most highly correlated to the changes in sevoflurane concentrations and the subsequent fluctuations in the SSEP waveforms. Each subfigure represents a different nerve: left ulnar (**a**), right ulnar (**b**), left posterior tibial (**c**) and right posterior tibial (**d**). Each subfigure displays the top features selected. The associated SSEP channel for each feature is shown in the left column in each figure.

Figure 6 displays the raw data points from each feature generated for each trial for all ten patients included in the study. To determine which features were more closely associated with the fluctuating end-tidal sevoflurane concentrations, the MRMR algorithm was used to calculate importance scores for each feature. These features were reduced to the top ten features demonstrating the most significant importance values. The results for each nerve after performing MRMR on all 132 features are summarized in Figure 7.

#### 3.2.1. Left Ulnar Nerve Dataset

Following MRMR for the feature section, the top ten features with the highest importance scores were selected for regression modeling. These features in order of importance were: the peak amplitude of the evoked response for channel CP4–Fpz, the latency valley of the evoked response for channel CS5–Fpz, the first principal component for channel CP4–CP3, the first principal component for channel CS5–Fpz, the third principal component for channel CP4–Fpz, the third principal component for channel CS5–Fpz, the peak or dominant frequency for channel CP4–CP3, the third principal component for channel CP4–CP3, the peak or dominant frequency for channel CP4–Fpz, and the valley amplitude for the evoked response for channel CS5–Fpz.

#### 3.2.2. Right Ulnar Nerve Dataset

Ten different features were identified from the MRMR calculation for the right ulnar nerve. The ten highest features selected for regression modeling in order were: the peak amplitude of the evoked response for channel CP3–CP4, the latency peak for the evoked response for channel CP3–CP4, the third principal component for channel CP3–CP4, the second principal component for channel CP3–Fpz, the peak or dominant frequency for channel CS5–Fpz, the peak or dominant frequency for channel CP3–CP4, the third principal component for channel CS5–Fpz, the area of the PSC for channel CS5–Fpz, and the valley amplitude for the evoked response for channel CS5–Fpz.

#### 3.2.3. Left Posterior Tibial Nerve Dataset

Only five features were assigned importance scores for the left posterior tibial nerve as all other features were not recognized as significant. Those five features selected for regression modeling in order were: the latency valley for the evoked response for channel CPz–Fpz, the first principal component for channel CPz–Fpz, the area of the PSC for channel CP3–Fpz, the third principal component for channel CPz–Fpz, and the third principal component for channel CP3–CP4.

#### 3.2.4. Right Posterior Tibial Nerve Dataset

Similarly, for the right posterior tibial nerve, MRMR assigned importance scores for only five features, as all others were insignificant. Those five features selected for regression modeling in order were: the latency peak for the evoked response for channel CPz–Fpz, the first principal component for channel CPz–Fpz, the second principal component for channel CP4–Fpz, the second principal component for channel CPz–Fpz, the third principal component for channel CPz–Fpz, the third principal component for channel CP4–Fpz, and the third principal component for channel CP4–CP3.

### 3.3. Modeling Results

Following feature selection for each nerve using MRMR, the models were trained and tested to determine which ML prediction model was the most accurate at predicting SSEP waveforms considering variable end–tidal sevoflurane concentrations. After training each model using the first eight patients’ SSEP data, the models were tested with the remaining two patients’ datasets. Summarized in Table 2 are the metrics for the highest-performing model found in each SSEP nerve dataset. To compare the performance of each model, RMSE values were compared. Lower RMSE values are representative of more accurate models.

Importantly, ML models and their associated parameters and hyperparameters contain much of the information about how the algorithm is performing. Implementing various parameter combinations during the learning, or training stage, is the process of coefficient optimization. By adjusting the coefficients, the model’s internal variables can continue learning and achieve maximum accuracy. After training, the quadratic SVM and coarse regression tree models tied for the greatest predictive performance. Table 3 shows the SVM ML model’s associated parameters and hyperparameters. This table displays the different information about the internal properties or components of the quadratic SVM algorithms utilized in this study. Furthermore, Table 4 presents the hyperparameters used in the quadratic SVM model. Table 5 presents the parameters used in the other top model, the coarse regression tree model. Table 6 subsequently shows the hyperparameters for the coarse regression tree.

#### 3.3.1. Left Ulnar Nerve

**Figure 8:**
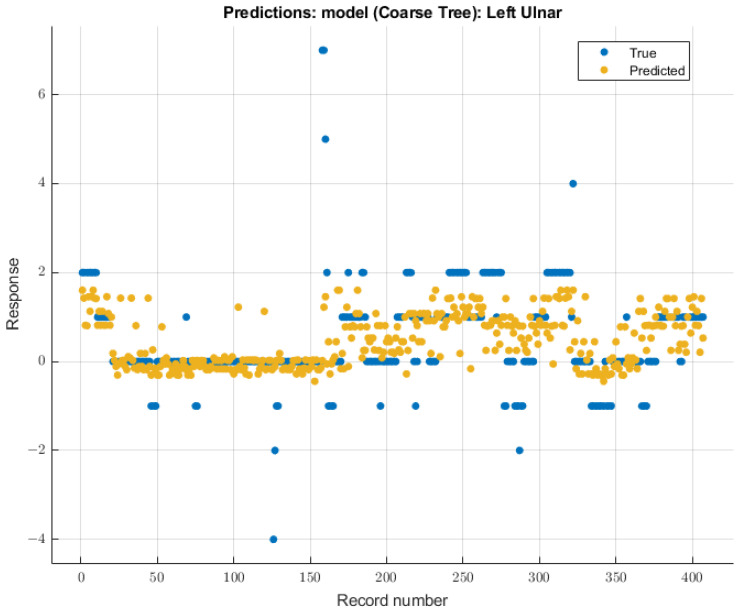
Left ulnar nerve prediction modeling results. The results of the prediction values for SSEP trials in the left ulnar nerve, which were generated by the coarse tree model. The true values are shown in blue, and the corresponding predicted values calculated by the model are shown in yellow.

The coarse tree was the model with the most significant validation score on SSEP waveforms for the left ulnar nerve due to changing end-tidal sevoflurane concentrations. The course tree model demonstrated an RMSE value of 0.91699 for validation using training data. The trained coarse tree model when tested on the testing dataset yielded an RMSE score of 2.3247. The modeling results for the left ulnar nerve are shown in Figure 8.

#### 3.3.2. Right Ulnar Nerve

**Figure 9:**
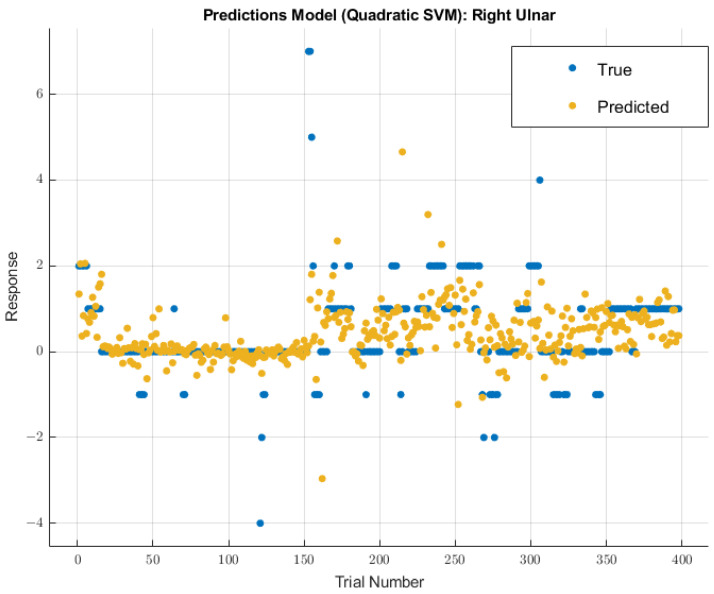
Right ulnar nerve prediction modeling results. The results of the prediction values for SSEP trials in the right ulnar nerve, which were generated by the quadratic SVM model. The true values are shown in blue, and the corresponding predicted values calculated by the model are shown in yellow.

A different model displayed greater future predictability for the right ulnar nerve than the coarse tree model used for the left ulnar nerve. The quadratic SVM model showed a validation RMSE value of 0.96147 using training data. Using the features selected from MRMR calculations, the RMSE value on the test data was 2.4047. The modeling results for the right ulnar nerve are shown in Figure 9.

#### 3.3.3. Left Posterior Tibial Nerve

**Figure 10:**
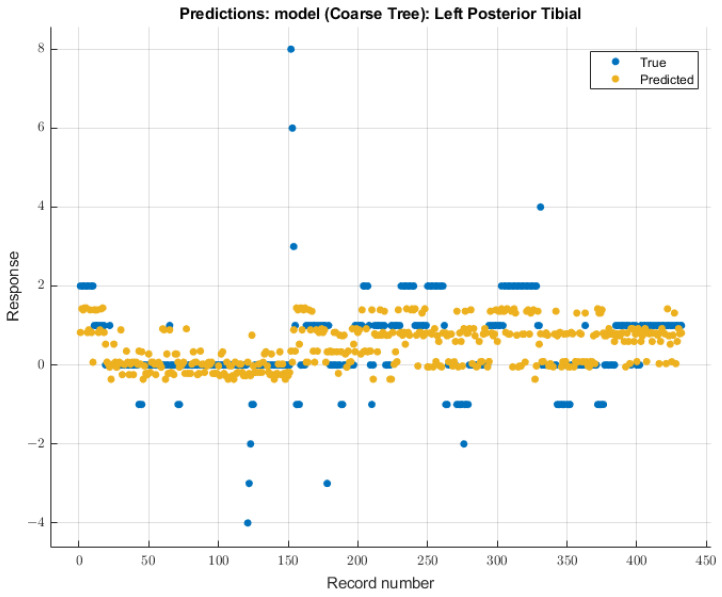
Left posterior tibial nerve prediction modeling results. The results of the prediction values for SSEP trials in the left posterior tibial nerve, which were generated by the coarse tree model. The true values are shown in blue, and the corresponding predicted values calculated by the model are shown in yellow.

For the left posterior tibial nerve, similar to the left ulnar nerve, the coarse tree model showed the most remarkable future predictability on SSEP waveforms. The course tree model demonstrated an RMSE value of 1.0229 for validation of the training data. During testing, this model showed an RMSE value of 2.244. The modeling results for the left posterior tibial nerve are shown in Figure 10.

#### 3.3.4. Right Posterior Tibial Nerve

**Figure 11:**
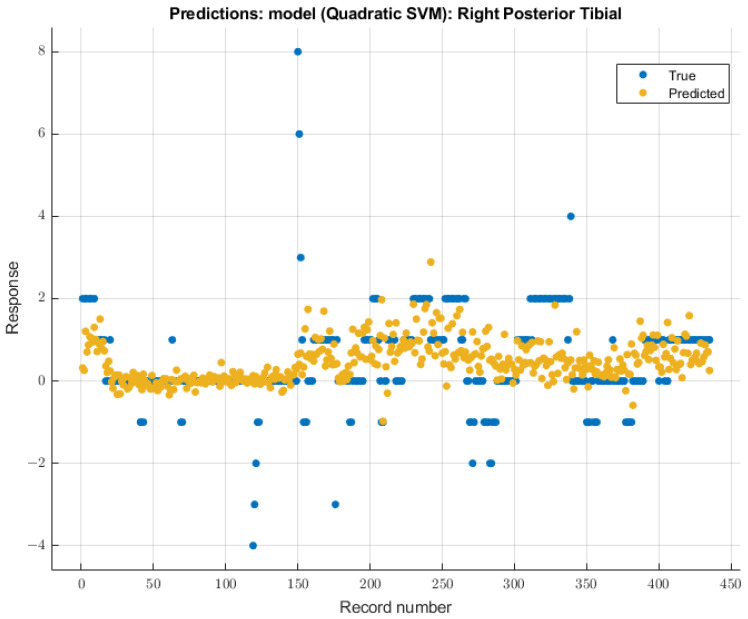
Right posterior tibial nerve prediction modeling results. The results of the prediction values for SSEP trials in the right posterior tibial nerve, which were generated by the quadratic SVM model. The true values are shown in blue, and the corresponding predicted values calculated by the model are shown in yellow.

As with the right ulnar nerve, the quadratic SVM model showed the most significant future predictability on the right posterior tibial nerve SSEP waveforms. During the validation of training data, this model showed an RMSE value of 0.99482. During the testing stage of this model, the RMSE value for the two patients’ data was 2.17. The modeling results for the right posterior tibial nerve are shown in Figure 11.

## 4. Discussion

This study examined the relationship between end-tidal sevoflurane concentrations and SSEP waveforms, with the primary objective of using signal processing and machine learning techniques to more finely characterize and predict SSEP waveforms. Signal processing techniques such as principal component analysis, time frequency analysis, and phase space curves, coupled with the analysis of evoked response amplitude and latency, enabled the conversion of complex SSEP datasets into measurable metrics. A specific set of features was generated from these various metric categories to expand the scope with which an SSEP waveform can be delineated. After using the minimum redundancy maximum relevance (MRMR) model for feature selection, a select group of SSEP features closely related to the change in end-tidal sevoflurane concentrations was identified. Interestingly, while the top feature for all the nerves monitored was derived from the evoked response, one of the three principal components relaying information about the waveform shape was also found to be among the top three highest-scoring features. This finding indicates that varying anesthetic concentrations can also affect the shape of SSEP waveforms as much as other quantifiable features describing the signal.

After identifying the features with the most significant importance scores, various machine learning models were then used to train datasets to determine the optimal model that can predict SSEP waveform form based on sevoflurane concentration. By applying the ulnar nerves’ top ten features and the posterior tibial nerves’ top five features to predictive models, it was found that the coarse tree and quadratic SVM models were the most effective in predicting future SSEP waveform distortions as a function of sevoflurane concentration. Root mean square error (RMSE) values were used to determine which ML method yielded the lowest error rate. Interestingly, the RMSE values for training the models showed less favorable predictive capabilities for the posterior tibial nerves, as compared to that of the ulnar nerves. This discrepancy may be due to the increased applied stimulation intensity (ranging from 0.04 to 0.06 amperes) necessary to generate robust evoked responses in the posterior tibial nerves, as compared to that of the ulnar nerves (ranging from 0.025 to 0.034 amperes), which is half the intensity applied. This finding suggests that an increased stimulation intensity maintains some capability to overpower the suppressive effects of sevoflurane on SSEP waveforms.

Anesthetic-driven alterations in SSEP signals are a well-known phenomenon. Volatile agents specifically tend to produce a dose-dependent increase in SSEP latency and a decrease in amplitude. Sevoflurane distinctively has less amplitude reduction compared to other volatile agents. The anesthetic cocktail of sevoflurane and remifentanil is preferred during SSEP monitoring due to sevoflurane’s rapid off effect and remifentanil’s short half-life, which together facilitates rapid on/off anesthesia [12,15,38]. However, this is not without controversy, as some articles have argued against sevoflurane having a significant effect on SSEP latency or amplitude [19,39]. Despite this, it is widely accepted that inhaled volatile anesthetics have a dose-dependent latency increase and decrease in amplitude [12,15,38]. Also, over recent years, the superiority of remifentanil with either propofol or sevoflurane has been highly discussed. The consensus is that sevoflurane has faster suppression and recovery of SSEP latency and more within-patient variability, with faster cessation of anesthetic effect and a more rapid awakening. In contrast, propofol has less impact on SSEP waveforms, but prolonged awakening from anesthesia and lengthy time to SSEP changes from concentration adjustments [38,40]. Ultimately, it has been shown that SSEPs can still be monitored safely despite the depressive effects of sevoflurane discussed above [12].

To improve the outcomes of the prediction models, future investigations must consider the limitations of the current study. First, developing accurate machine learning models is a significant challenge due to the requirement of large data pools for generating reliable prediction metrics. In this case, only ten patients were used for feature generation, of which eight were used for training and the remaining two for testing. As a result, the availability of data was a significant hurdle. Secondly, while sevoflurane is known to suppress SSEP waveforms in a dose-dependent manner, its variability during the procedure can hinder the establishment of a clear correlation. In these cases, the variability mainly occurs at the beginning or end of the operations, specifically during the transition from induction to maintenance anesthesia, and from asleep to awake at the end of the case when the inspired sevoflurane is decreasing. This indicates that there are only two small windows within each case where the sevoflurane end-tidal concentrations can be more variable than during the operation’s middle portion. Third, this study did not account for the changes in other variables during the operation, such as oxygenation, blood pressure, heart rate, or other administered medications. To improve the accuracy of the prediction models, fluctuations in variables associated with hemodynamic parameters, signal disturbance caused by positioning or instrumentation, and other sources, such as preexisting deficits, need to be considered. Ultimately, to generate the most robust model, a collaborative effort between multiple institutions to collect a larger dataset while controlling for intraoperative variables is of the utmost importance. Additionally, the lack of standardization of IONM protocols among different institutions and IONM technicians makes it challenging to analyze IONM data. This lack of standardization has been recognized for some time and, in large part, is one of the foundational reasons to use predictive machine learning tools to further develop neuromonitoring capabilities [41].

Deviations from baseline SSEP signals are important to surgeons, anesthesiologists, and, most importantly, to patients. The primary purpose of IOMN monitoring is to detect any impending sign of neurologic injury early with the goal of identifying and reversing the original insult before a permanent deficit occurs. While surgical factors leading to signal alterations are deemed most important, numerous other factors such as hemodynamic parameters, patient positioning, monitoring equipment malfunction, and medications are important considerations to avoid treating a false alarm [38]. When deviations from baseline SSEPs occur, it takes troubleshooting and communication by all operating room personnel to identify the cause. This added time to the procedure can distract the surgeon and prolong the operation. As a result, having the ability to predict in real-time the different etiology related waveforms alterations can significantly benefit all parties involved. This study is a small step towards that goal. It applies machine learning strategies to quantify and predict the effect of one particular factor, namely sevoflurane on SSEP waveforms. The results demonstrate the need to expand the variables and metric categories for analyzing IONM signals. Subsequent investigations will involve other anesthetic agents, non-surgical factors, and additional vital sign parameters to delineate the various effects of these variables on SSEP waveforms. Given the limited dataset available, the primary goal of this study has been to explore how the principles of machine learning can be applied to the analysis of a complex set of neurophysiologic data, rather than to generate meaningful conclusions regarding the effects of volatile anesthetics on SSEP. Future studies with a larger dataset, using the principles established from this research, are expected to provide more meaningful conclusions.

## 5. Conclusions

The findings of this study are expected to contribute to the advancement of our understanding of neuromonitoring and its potential, and to pave the way for the development of new diagnostic and therapeutic tools for neurological disorders. This research provided preliminary evidence that ML models can be used to predict SSEP signals alterations when provided with non-surgical factors and that the integration of non-surgical factors can improve the predictive accuracy of IONM ML models. The findings provide a glimpse into the future of the healthcare industry, as clinicians may one day use more advanced models to anticipate the outcome of surgical procedures and take proactive measures to prevent adverse events. As more development is made to these systems, more data about non-surgical factors can be added to these models. This will allow for more robust predictive capabilities and lead to the development of the next generations of IONM systems. As such, the outlook for application of machine learning strategies to intraoperative neuromonitoring is promising. This study shows that it is possible to break down the complex SSEP waveforms into measurable and definable features, which allows for a more complex and nuanced analysis of this interesting phenomenon, elevating it from just simple analysis of amplitude and latency of evoked responses. The use of human data is also of significant relevance to clinical application. By presenting a detailed protocol to predict the effect of a volatile anesthetic on SSEP waveform, the findings highlight the potential for machine learning applications to advance patient safety and care in spine surgery.

## Figures and Tables

**Table 1:** Patient demographics table.

Patient Number	Gender	Age	Procedure
1	Male	62	Lumbar Decompression and Fusion
2	Male	74	Lumbar Decompression and Fusion
3	Male	60	Lumbar Decompression and Fusion
4	Female	52	Lumbar Decompression and Fusion
5	Male	51	Lumbar Decompression and Fusion
6	Male	50	Lumbar Decompression and Fusion
7	Female	63	Lumbar Decompression and Fusion
8	Female	66	Thoracic Decompression and Fusion
9	Male	49	Lumbar Decompression and Fusion
10	Male	61	Lumbar Extradural Tumor Excision and Fusion

**Table 2:** Model parameters and performance.

	Left Ulnar	Right Ulnar	Left Posterior Tibial	Right Posterior Tibial
Model	Coarse Tree	Quadratic SVM	Coarse Tree	Quadratic SVM
RMSE (Validation)	0.91699	0.96147	1.0229	0.99482
R–Squared (Validation)	0.23	0.09	0.03	0.1
MSE (Validation)	0.84086	0.92442	1.0464	0.98968
MAE (Validation)	0.57944	0.5955	0.66129	0.62981
Prediction Speed (Observations/sec)	5300	27,000	23,000	27,000
Training Time (Sec)	12.712	5.0486	2.1419	2.4806
Test Results (RMSE)	2.3247	2.4047	2.244	2.17

**Table 3:** Support vector model parameters.

Property	Value
Box Constraint	0.7413
Cache Size	10^3^
Caching Method	Queue
Clip Alphas	1
Epsilon	0.0741
Gap Tolerance	10^−3^
Iteration Limit	10^6^
Kernel Function	Polynomial
Kernel Scale	Auto
Solver	SMO
Standardize Data	1
Save Support Vectors	1
Version	2
Type	Regression

**Table 4:** Support vector model hyperparameters.

Property	Value
Preset	Quadratic SVM
Kernel Function	Quadratic
Kernel Scale	Automatic
Box Constraint	Automatic
Epsilon	Automatic
Standardize Data	Yes

**Table 5:** Regression tree model parameters.

Property	Value
Split Criterion	MSE
Minimum Parent	72
Minimum Leaf	36
Maximum Splits	406
NVar To Sample	All
Merge Leaves	On
Prune	On
Prune Criterion	MSE
QEToler	10^−6^
NSurrogate	Off
Maximum Cat	10
AlgCat	Auto
Predictor Selection	All Splits
Use Chi-Square Test	1
Version	3
Method	Tree

**Table 6:** Regression tree model hyperparameters.

Property	Value
Preset	Coarse Tree
Minimum Leaf Size	36
Surrogate Decision Splits	Off

## Data Availability

Data sharing not applicable.
